# *“I tell them to have Sumud, have Sabr, for your children, for yourself”*: Culturally informed resilience and humanitarian gaps among Syrian refugee women in Greece

**DOI:** 10.1177/17455057261473454

**Published:** 2026-08-20

**Authors:** Karen Youssef, Gulnaz Anjum, Halina Grzymała-Moszczyńska

**Affiliations:** 1 6305 Department of Psychology, University of Oslo, Oslo, Norway; 2 Department of Psychology, 6305 University of Oslo, Oslo, Norway; 3 Institute of Psychology, 357321 Ignatianum University in Krakow, Krakow, Poland

**Keywords:** resilience, Sumud, Sabr, Syrian refugee women, life in limbo, intergenerational resilience, NGO interventions

## Abstract

**Background:**

The journey in exile for Syrian refugee women is filled with constant uncertainties as they balance motherhood, grief, trauma, discrimination, and prolonged asylum procedures. While humanitarian organizations repeatedly rely on “resilience” frameworks, our work focuses on Syrian communities’ native language, history, lived realities, and cultural idioms such as Sumud (steadfastness) and Sabr (patience).

**Objective:**

This study explores how Syrian refugee mothers living in Lesvos define, practice, and transmit resilience to their children throughout their journey, and examines the extent to which NGO interventions align with their culturally informed understandings of resilience.

**Methods:**

Eight Syrian refugee women residing in Kara Tepe camp participated in semi-structured interviews conducted in Arabic. Data were analyzed using reflexive thematic analysis within an interpretivist and decolonial framework, emphasizing women’s own voice, language, and narrative.

**Results:**

Four overarching themes appeared. *(1) Becoming Through Sabr* showed that women described their resilience primarily through Sabr; a cultural and spiritual form of patient endurance. Resilience was both a necessity and a way of reclaiming autonomy. *(2) Roots without Soil* captured how motherhood constantly transforms in exile as many become solo mothers. Intergenerational resilience was marked by storytelling, future promises, and spiritual reassurance. *(3) The Paradox of Sanctuary* reflected the dire conditions they faced along their journey and the contradiction between the promised haven and the reality of “sanctuary”. *(4) NGO Support and Gaps* demonstrated that women appreciate kind treatment, yet significant gaps still persisted in addressing ecological needs, cultural and gender-specific adaptations, and structural alignment.

**Conclusion:**

Resilience for Syrian mothers was embodied through Sabr, maternal responsibility, family bonds, and communal solidarity. Their narratives call for humanitarian interventions co-created with the community and that address the gap between humanitarian policy and on-the-ground implementation.

## Plain language summary

Millions of Syrian women have been forced to leave their homes because of war. Many now live in refugee camps in Europe while they wait for decisions about their future. Aid organizations often describe these women as “resilient,” but their programs rarely draw on the women's own ideas, language, and beliefs about staying strong. In this study, we wanted to understand strength and survival from the women's own point of view. We interviewed eight Syrian refugee mothers living in a camp on the Greek island of Lesvos. We spoke with them in Arabic and focused on their own words and meanings. The women drew their strength from faith, from their children, and from their sense of community. Many described their strength using the Arabic word “Sabr,” which means active, patient endurance. This mattered to them even more than “Sumud,” a word for steadfastness that we had expected to hear. The mothers cared for their children under very hard conditions, including unsafe shelter, poor food, and a long, uncertain wait for asylum. Our findings suggest that the burden of staying strong should not rest on the women alone. Humanitarian organizations and policymakers should listen to refugee women's own understandings of endurance and offer support that matches their real needs. Practical, respectful, and culturally aware help can make a meaningful difference to mothers and children living through displacement.

## Introduction

Before war unsettled every certainty and before borders redefined life as they knew it, Syrian women lived within worlds anchored by family, community, language, and faith. Everyday life was shaped by many intersecting elements of their identity; holding households together, caring for children, pursuing dreams, and sharing meals and memories with their community. These worlds were ruptured abruptly when the Syrian war erupted in 2011.^1^ Over half of the country’s population were displaced and millions were forced to seek refuge in neighboring countries such as Turkey, Lebanon, and Jordan, many imagining their stay as temporary.^2^ However, the situation worsened and the possibility of long-term solutions remained unknown, increasing numbers of Syrians attempting to seek refuge in Europe. The path towards Europe was often taken from Syria, through Turkey, and across the Aegean Sea to the Greek islands, with Lesvos island becoming one of the primary points of arrival.^3^ Before attempting to cross the sea, 3.6 million Syrians sought refuge in Turkey. Yet, the years spent in Turkey were marked by economic exploitation, social discrimination, and lack of protection, forcing some to risk the sea in search of safety within the EU.^4^

The Aegean Sea crossing became its own ordeal, defined by overcrowded rubber boats, unpredictable waves, and violent coast guard pushbacks, where many lives were lost before reaching land.^5,6^ Those who managed to survive landed on Lesvos, a small island that in 2015 alone, received approximately 379,000 arrivals; an overwhelming number relative to its population of about 86,000 residents.^7^ Reception facilities on the island are reported as inadequate, generating severe overcrowding, unsanitary conditions, and security risks.^8^ Women and children face life-threatening challenges in the camp including risks of sexual violence, lack of privacy, and poor access to healthcare.^9,10^ As the situation worsened, civil society stepped in to fill critical gaps. Alongside major NGOs operating on Lesvos, several ad hoc grassroots organizations were created to provide immediate humanitarian relief, translation, clothing, food distribution, and psychosocial support. Volunteers often arrived with little to no professional humanitarian backgrounds yet responded rapidly wherever needed and whenever formal systems lagged.^11^

In the early months of 2025, this landscape shifted once again as several EU member states paused the processing of Syrian asylum applications following the political changes in Syria associated with the fall of the Assad regime.^12^ For refugees already living in Kara Tepe camp on Lesvos, the promise of sanctuary was fractured, leaving them in a prolonged state of legal limbo as they are unable to return home, move forward, or rebuild their lives with stability.

The journey of Syrian refugees has undoubtedly gained global attention. Their suffering stood at the center of humanitarian discourse, as scholars and humanitarian actors have documented their journey in exile through narratives of trauma, forced migration, and the psychological impacts of seeking asylum.^13^ These narratives have played an important role in mobilizing resources and raising awareness. Yet, the repetition of this narrative has also led to a normalization of their pain. Syrians have become clustered into the broader category of “refugees”, reducing the distinctive dimensions of their identity, culture, strength, and survival and masking the way they understand and practice continuity and resilience.^14^

**Figure 1.**
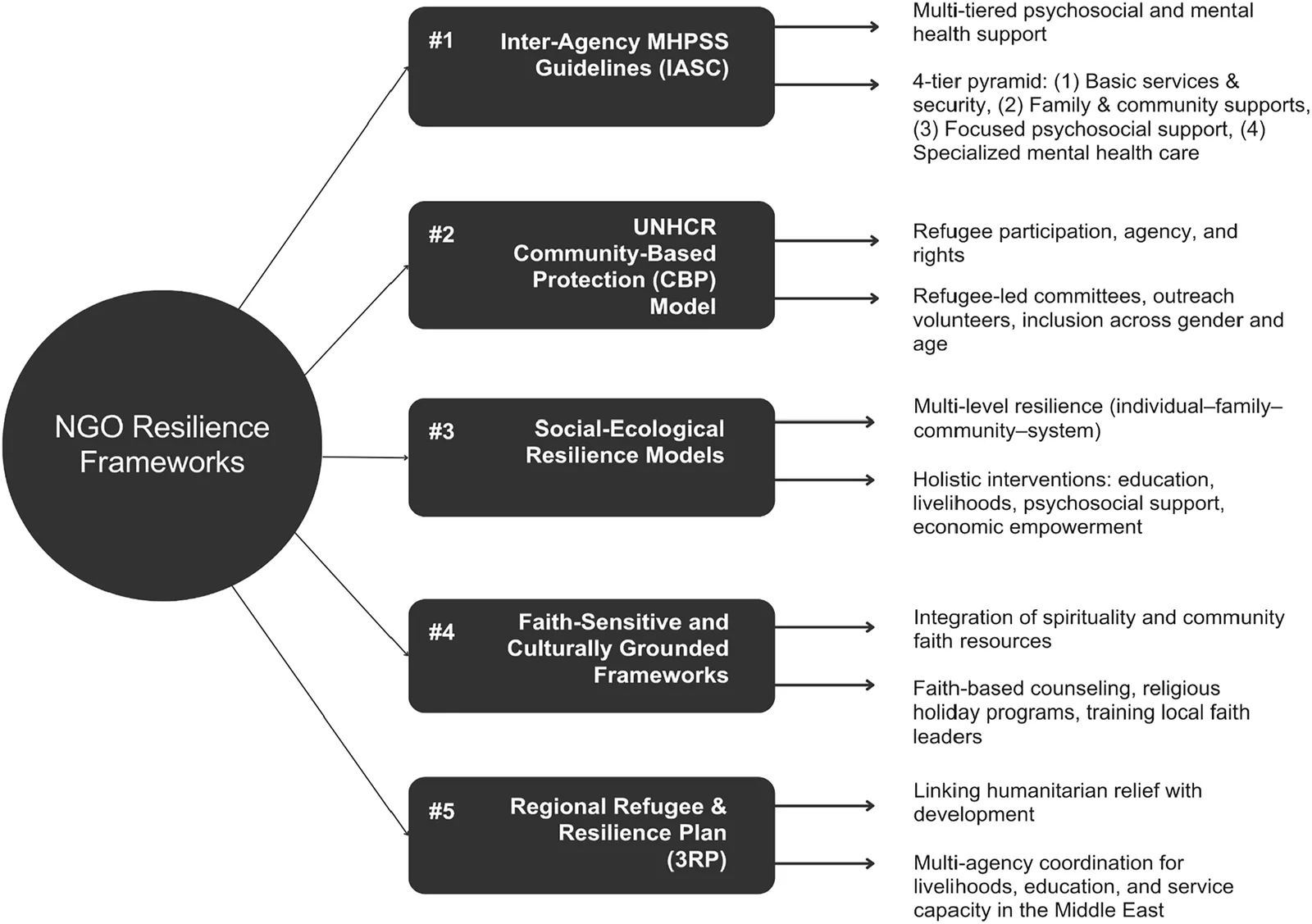
NGO resilience-building frameworks and their core components.^19–23^

These realities raise the central concern of this study: how resilience is understood and enacted by Syrian refugee women themselves, and how closely humanitarian frameworks reflect those understandings. Resilience has become a dominant framework in humanitarian and development discourse, shaping how NGOs and UN agencies understand individuals’ and communities’ capacities to withstand, adapt to, and recover from crises while maintaining dignity and well-being. Its global rise reflects a broader shift toward multi-dimensional, sustainability-oriented approaches to aid and adaptation, especially in contexts of severe precarity.^15^ The core components of the resilience frameworks most commonly applied by humanitarian organizations are summarized in Figure 1. As much traction as the concept of resilience has gained, it has equally become a buzzword that is frequently invoked in the face of adversity but rarely interrogated. Across humanitarian settings, resilience is often celebrated as a trait or as the marker of a “successful recovery story”.^16^ Much of the evidence base informing these resilience-building programs has been shaped by research and practice originating in Western or high-income contexts, where resilience discourse has often emphasized rapid recovery, autonomy, and self-reliance. This is not to suggest that endurance in the face of hardship is absent elsewhere. Women across many cultures and histories have long drawn on their own traditions of strength. The concern, rather, is that the conceptual vocabulary embedded in dominant humanitarian frameworks can privilege particular cultural assumptions about what resilience should look like.^17^ Such policies reflect “the logic of traditional humanitarian aid,” which removes responsibility from systems and institutions and places the burden of coping onto the individuals who must endure adversity.^18^

To give the power of narration back to the community and understand how Syrians “remain” amid precarity, attention must be directed to the power of language. This paper acknowledges that concepts cannot always be translated effortlessly across linguistic and cultural boundaries, and recognition of value and influence of the native language cannot be overlooked but only embraced.^24^ Hence, to understand Syrian resilience, it is important to recognize that the term “resilience”, in which a variety of frameworks have been built upon, lacks a direct equivalent in Arabic that captures its precise meaning. The closest cultural expression to resilience in the Arabic language is “Sumud”, which directly translates as “steadfastness”.^25^ This word has gained historical and cultural traction in the Palestinian context after 1967 as it became central to the narrative of resistance against Israeli occupation.^26^ Beyond the Palestinian context, the meaning of Sumud has circulated in other Arab-speaking neighboring communities as resilience.^27^ While Sumud was addressed in different cultural contexts within the region, there is little to no research on Sumud or any culturally-specific ideologies of resilience within the Syrian community.^28^

## Theoretical framework

Understanding Syrian refugee women’s experiences requires an approach that moves beyond singular or universal models of resilience. Research with communities that are constantly on the move, facing unpredictable states of uncertainty, and new challenges require a complex lens that can adequately capture the layered realities of their experiences in displacement. NGO interventions and social-ecological models do important work in addressing systemic vulnerabilities. They tend to give less attention to how some forms of knowledge come to count more than others when resilience is defined. To build on these models, this study adopts an integrated theoretical framework grounded in decolonial psychology and intersectionality, while engaging the concepts of limbo and culturally embedded idioms such as Sumud as interpretive tools. Within this framework, decolonial psychology serves as the primary analytical lens. It helps to keep local and cultural understandings of resilience in view alongside established models. Intersectionality and the Life in Limbo framework function as supporting lenses that sharpen attention to overlapping inequalities and to the effects of protracted uncertainty. Sumud and Sabr are treated as the cultural and linguistic entry points through which the women themselves articulate endurance.

Decolonial psychology draws attention to how resilience research and humanitarian practice have often been shaped by knowledge produced in Western and high-income settings.^29,30^ Mainstream models often frame resilience in terms of self-reliance, and can give less weight to collective, cultural, and relational forms of endurance.^31^ In displacement contexts, a decolonial lens helps to bring culturally grounded idioms such as Sumud into focus. These idioms frame survival as a situated, relational, and at times political practice within ongoing disruption, rather than as a simple, linear recovery. From this perspective, resilience is better understood as more than a psychological trait. It is also connected to questions of recognition and epistemic justice.

Intersectionality further sharpens the analysis by illuminating how overlapping systems of oppression shape refugee women’s lived experiences. Crenshaw’s work highlights that race, gender, class, and other identity markers cannot be understood in isolation but intersect to produce complex forms of marginalization.^32^ Within refugee contexts, this framework reveals how vulnerabilities are compounded by legal status, ethnicity, motherhood, and displacement, while also recognizing that women demonstrate agency and resilience across these intersecting axes.^33–35^ For instance, the risk of gender-based violence cannot be disentangled from asylum policies, racial profiling, and family separation.^9,36^

While this study is primarily guided by the mentioned perspectives, it also draws selectively on concepts of limbo and culturally grounded idioms of resilience to enrich interpretation. The Life in Limbo framework, while not adopted as a central model, offers an important conceptual backdrop in this research particularly due to the research being situated in a period where asylum seeking procedures were paused for Syrians. The Life in Limbo framework describes the psychological consequences of prolonged uncertainty, where asylum seekers remain suspended between belonging and exclusion.^37^

**Figure 2.**
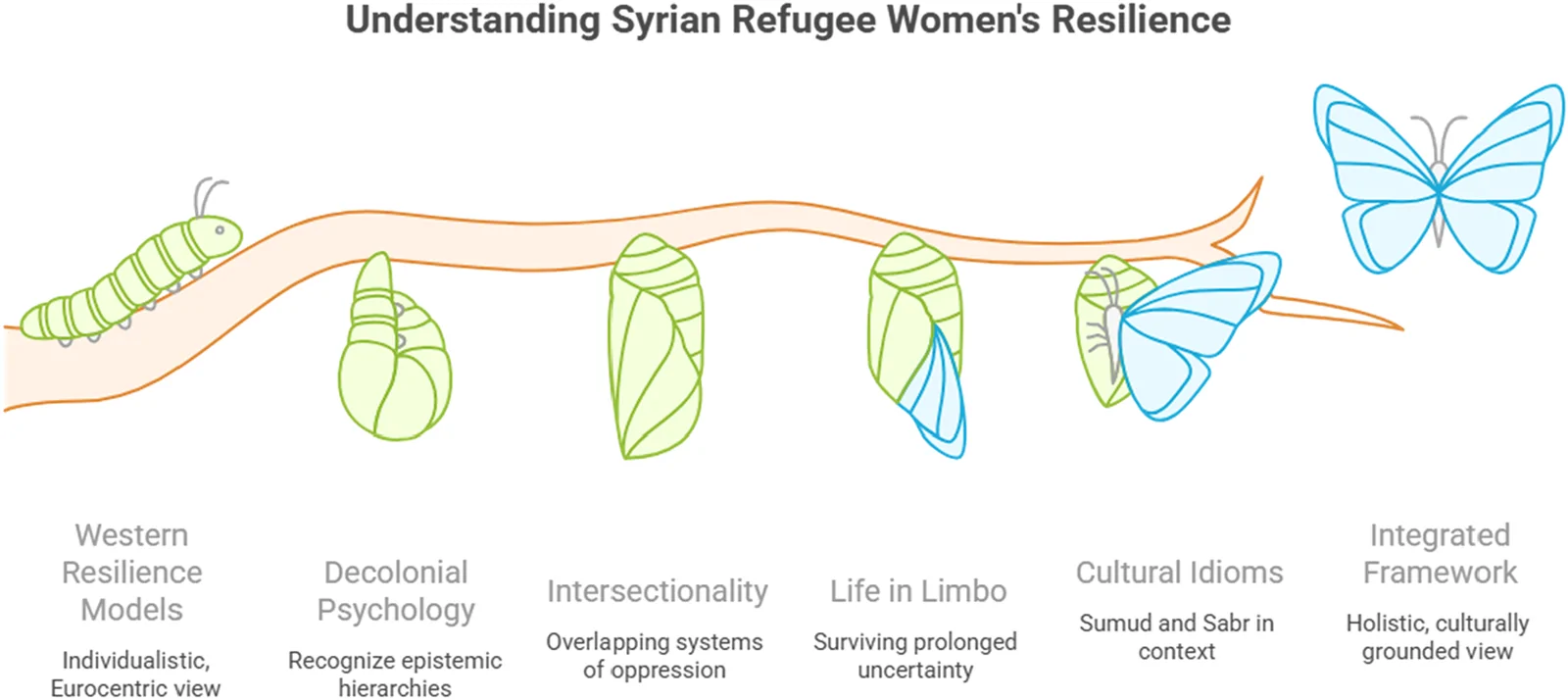
Integrated framework for understanding Syrian women’s resilience. *Note*. The conceptual elements shown (Western resilience models, decolonial psychology, intersectionality, Life in Limbo, and cultural idioms such as Sumud and Sabr) are analytical lenses that inform this study. The butterfly imagery is used as a heuristic for integration and is not intended to imply a linear or stage-based developmental progression. Consistent with the study’s argument, resilience is understood here as fractured, cyclical, and non-teleological.

Similarly, this study adopts Sumud, not as a theoretical lens, but rather as a cultural, linguistic, and analytical entry point into Syrian refugee women’s own conceptualizations of resilience. While “resilience” as a psychological construct lacks a direct equivalent in Arabic that captures its full psychological connotations, Sumud provides a culturally resonant framework for understanding survival, spirituality, and collective responsibility.^25,38^ Taken together, these pillars provide a robust framework for situating Syrian refugee women’s narratives of displacement within both structural conditions and cultural epistemologies. Figure 2 shows an integrated theoretical model.

## Current study

This study uses Sumud as an analytical lens to examine the cultural and linguistic foundations of Syrian resilience, focusing on how refugee mothers and children draw on culturally rooted practices of endurance amid displacement. Grounded in an intersectional and decolonial framework, it highlights the ways resilience is shaped by culture, identity, race, structural constraints, and material precarity, aspects largely overlooked in dominant humanitarian narratives.

As resilience discourse expands within humanitarian policy, existing frameworks often miss the cultural idioms and collective practices that define Syrian refugee women’s lived realities. By centering Syrian voices and applying Sumud to move beyond Western-centric models, this study explores how resilience is defined and enacted within the community, particularly among mothers and children in Lesvos, Greece. The aim is to generate insights that inform more contextually grounded humanitarian practice, guiding NGOs and policymakers toward interventions aligned with Syrian families’ values, needs, and aspirations. The project is guided by three central questions:

• How do Syrian refugee women define, experience, and practice “Sumud” along their journey (before leaving the homeland, during, and future)?
• How do these women pass on Sumud’s ideological value to their children and the younger generation?
• How do NGOs’ resilience-building interventions align with that of the communities’ culturally informed resilience?

## Methods

This study adopted a qualitative research design to explore how Syrian refugee mothers and children define, experience, and practice Sumud throughout their displacement journey. A qualitative approach was used for an in-depth understanding of lived experiences, social meanings, and cultural interpretations.^39^ Data were analyzed using reflexive thematic analysis due to the flexibility and its emphasis on researcher’s critical self-awareness of their role, assumptions, positionality, and influence throughout the research process.^40^ This approach allowed deep engagement with the data and center the focus on women’s voices and interpretations.

### Participants

Although this project initially aimed for recruiting 10 participants, the present study involved eight Syrian refugee women, all mothers aged 18 years and older, residing in Kara Tepe camp on the island of Lesvos, Greece. The decrease in number was caused by audio recording complications and researcher’s limited time on the island due to visa restrictions. The participants varied in marital status, asylum-seeking status, and length of displacement. This heterogeneity allowed for a more comprehensive exploration of Sumud across different phases of migration and motherhood. Participant demographics are illustrated in Table 1.

**Table 1.** Summary of participants.

Pseudonym	Age range	Marital status	Children	Asylum stage
1/Layla	25–34	Widowed	2 (all with her)	Pending/Unknown
2/Nour	25-34	Married (husband in EU)	5 (3 with her; 2 in Turkey)	Pending/Unknown
3/Amal	35-44	Widowed	2 (all with her)	Pending/Unknown
4/Nadine	25-34	Married (husband in EU)	4 (all with her)	Accepted/awaiting reunification
5/Zahra	40–45	Married (husband in Syria)	2 (1 with her, other in Syria)	Pending/Unknown
6/Farah	18-24	Married (Husband present)	1 (with her)	N/A
7/Sarah	25-34	Married (Husband in EU)	5 (all with her)	Pending/Unknown
8/Amar	25-34	Separated	2 (both in Syria)	Pending/Unknown

*Note. *All names are pseudonyms. Age ranges and asylum stages reflect participants' circumstances at the time of data collection (February–May 2025), during the suspension of asylum processing for Syrian applicants in Greece. N/A = not applicable.

### Procedure

#### Recruitment

Participant recruitment took place through a local community center, where the first author worked in the women’s space and supported as an Arabic translator and cultural mediator. These roles, along with shared language, facilitated rapport and trust, enabling recruitment through convenience and snowball sampling. Additional participants were referred through existing networks, which increased access but may have introduced bias by drawing primarily from closely connected social circles.^41^ Participants were initially briefed in person or through phone in regards to the study’s procedure. Before the interviews, participants carefully read the information letters in Arabic explaining the project’s purpose. The consent forms were signed and were provided in both English and Arabic. Eligibility was limited to adult women (aged 18 years or older) of Syrian origin who were mothers and who were residing in the Kara Tepe camp on Lesvos at the time of data collection. Women were excluded if they were under 18 years of age, were not mothers, or were unable to take part in an interview conducted in Arabic. Data were collected during a single fieldwork period between February – May 2025.

### Interviews

The interviews took place in person at Terra Psy; a mental health NGO operating within Parea, in spaces outside of the camp to ensure privacy and a safe environment. Interviews were scheduled with sensitivity to Ramadan fasting hours to ensure comfort and privacy for participants. Psychologists and trained volunteers were available on site to assist with the participant’s children during the interviews to ensure mothers could engage in the interview with minimal disruption. The semi-structured interviews lasted approximately 60 minutes. The interview guide covered five domains:

1. Sumud as an abstract concept
2. Sumud in practice
3. lived experiences of Sumud
4. intergenerational transmission of Sumud
5. NGO interventions and resilience-building frameworks

All interviews were conducted in Arabic; the native language of both participants and researcher, to enable accurate expression and cultural authenticity.^24^ Audio recordings were transcribed and translated into English. Transcription and translation were carried out by the first author, a native Arabic speaker who had also conducted the interviews. This allowed dialect, tone, and idiom to be interpreted in the context in which they were spoken. Culturally specific terms whose meanings are not fully captured in English, such as Sumud, Sabr, Alhamdulillah, and Inshallah, were retained in Arabic and accompanied by explanatory glosses rather than replaced with approximate equivalents. Where expressions were idiomatic, translation prioritized conveying their intended meaning over a word-for-word rendering, in line with a meaning-based approach to cross-language qualitative research. Each participant received 20-euro compensation, provided through the researchers’ research funds.

### Reflexivity

#### Epistemological standpoint

This study adopts an interpretivist epistemology, viewing knowledge as socially constructed and context-dependent, an approach well suited to research with displaced populations whose experiences are inseparable from precarity, power, limbo, and structural marginalization. Anchored in decolonial psychology, it challenges Eurocentric knowledge hierarchies and centers the voices and lived realities of Syrian women. This epistemological stance aligns with reflexive thematic analysis, which recognizes the researcher’s active role in interpreting and co-constructing meaning and requires reflexivity rather than claims of neutrality.

Findings aim not for positivist generalizability but for transferability through contextualized insights that illuminate resilience in settings of forced migration. This standpoint underpins the study’s contribution: reframing resilience research as an endeavor that listens to, learns from, and validates displaced women’s own frameworks of endurance amid ongoing limbo and structural violence.

#### Researchers' positionality

The first author (KY) is a Lebanese woman who migrated to Europe and worked in the NGO sector on the island, positioning her between the Western humanitarian system and the Syrian refugee community. This liminal role created both proximity and distance: participants felt culturally comfortable with her, while her NGO affiliation called for ongoing reflexivity about power, gratitude, and critique. She built trust through communal activities and informal conversations about shared cultural references, drawing on her earlier work with Syrian refugees in Lebanon. The second author (GA) is a Pakistani woman working in European academia, whose Global South background and experience of navigating racialized institutional spaces heightened her sensitivity to voice, belonging, and epistemic inequality while guarding against over-identification. She remained attentive to the histories of displacement that bring Syrian families to Europe and encouraged the team to support a decolonial approach that keeps participants' voices, meanings, and agency at the center. The third author (HG-M) is a professor with expertise in the psychology of migration and cross-cultural research, and she recognizes that her training and position within European academia shaped her reading of the data. Together, these intersecting migrant and academic perspectives shaped a culturally attuned and ethically reflexive approach to the research.

#### Ethical consideration

Ethical approval for the study was obtained from the Research Ethics Committee at University of Oslo, and the processing of personal data was assessed and approved by the Norwegian Agency for Shared Services in Education and Research (Sikt) (Project Reference Number: 34537849; project duration: 20.12.2024 - 31.07.2025). All participants provided written informed consent with assurances of confidentiality, anonymity, and the right to withdraw. Pseudonyms were assigned and identifying details removed during transcription. Given the sensitivity of refugee experiences, interviews were conducted in private, safe settings by a researcher trained in active listening and crisis response. Participants were reminded they could pause or stop at any time, and on-site psychologists were available if support was needed. These safeguards ensured that emotional well-being remained a central priority throughout the study. Please see Appendix A for Information Letter and Consent Form; Appendix B for Interview Guide, Appendix C for additional documentation on Research Ethics Committee Approval, and Appendix D for SIKT Data Management Plan.

#### Analysis

Recordings were transcribed, pseudonymized, and translated into English. Data were analyzed using Braun and Clarke’s six-phase reflexive thematic analysis: (1) familiarization with the data, (2) generating initial codes, (3) searching for themes, (4) reviewing themes, (5) defining and naming themes, and (6) producing the report.^40^ Coding proceeded primarily inductively, staying close to participants’ own words and meanings, while the analysis remained theoretically informed. Existing scholarship on resilience, decolonial psychology, and Sumud sensitized the research team to particular patterns in the data. The analytic orientation is therefore best described as abductive, moving iteratively between participants’ accounts and theory rather than testing predefined categories. Reflexive notes and coding decisions were documented throughout to ensure transparency. Given the in-depth, idiographic focus of the study and the small purposive sample, the adequacy of the data was considered through the lens of information power rather than statistical saturation. The sample carried relatively high information power because participants were closely matched to the study aim, the interviews were detailed, and the analysis was theoretically grounded. Recruitment and analysis continued until the team judged that the accounts were yielding rich and recurring patterns and that additional interviews were unlikely to alter the developing thematic structure in substantive ways. With eight participants, no claim of complete thematic saturation is made. Reporting of the study follows the Consolidated Criteria for Reporting Qualitative Research (COREQ),^42^ and the completed checklist is provided as Supplementary Material.

## Results

In this section we present the analytical findings of the study. The analysis privileges the women’s voices and culturally grounded idioms of resilience to illustrate how Sumud (steadfastness) is lived, narrated, and transmitted across generations. Women’s accounts highlight resilience as a fluid and situated practice, often compelled by structural precarity and sustained through faith, motherhood, and collective endurance. Each theme, along with its subthemes, illustrates different dimensions of how resilience is culturally defined, relationally enacted, and structurally constrained in the lived realities of Syrian refugee women. Throughout the analysis, extracts presented in quotation marks or as indented text are participants’ own words, while the surrounding text reflects our analytic interpretation of those accounts.

### Theme 1. Becoming through Sabr: Syrian womanhood and the cultural fabric of Sumud

Sumud is often conceptualized as an unwavering and heroic stance against adversity. For Syrian refugee women, however, it was neither fixed nor constant; it shifted across time, space, and circumstance. It appeared in moments of purpose, yet fractured under the weight of forced choices, repeated losses, and structural constraints. This theme explores how women defined and embodied Sumud through survival, adaptation, and defiance.

#### Subtheme 1.1. No alternative but survival

The women’s testimonies revealed Sumud as a non-negotiable demand in the face of “life or death”. Leaving their homeland and crossing the sea was repeatedly described as the “journey of death”, leaving no space for vulnerability:

When you cross the sea, you have to force yourself to be strong. Everyone who made it here had to have Sumud. If you’re not strong, you'll be destroyed. (Amar, Participant 8)

The idea of “life or death” extended beyond physical danger to include an inhumane life under poverty, hunger, and erosion of basic rights. Sumud became essential for a dignified life. Amal echoed, “There’s nothing left for both me and my children…even if my child asks for a chocolate bar, I can’t.”

Strength was understood not only as an individual determination but as a cultural and collective responsibility, particularly towards family:

They [Syrians] are hardworking…They worry about their family. They think if I don’t work today, how will they eat? (Nadine, Participant 4)

Across all interviews, family and children emerged as the primary source of strength and motivation. The inevitability of hardship, coupled with a strong cultural duty to protect their family, shaped how women perceived and embodied Sumud.

#### Subtheme 1.2. The language of hope that endures

In many stories, language became a shelter; a living expression of faith, culture, and survival. When topics of war, loss, or personal struggles resurface; the women’s statements often ended with gratitude. These expressions carried emotional weight, allowing grief and hope to coexist:

In Syria, there was fear and horror…We left a few days before an airstrike destroyed our apartment. We would have died…Alhamdulillah we made it out alive. (Sarah, Participant 7)

Across all eight interviews, Alhamdulillah (thanks be to God) and Inshallah (God willing) appeared consistently. Alhamdulillah, often used as thankfulness, was mentioned 33 times across the interviews. Inshallah, used to express hopefulness, appeared 24 times. While both terms are religious in essence, their use was also cultural, revealing the influence of spiritual grace and hope in facing adversities.

Among these expressions,“Sabr” carried the deepest resonance. Mentioned 40 times across seven interviews, Sabr – often translated as “patience”, holds a far more layered meaning in Arabic. Nour even insisted that Sabr was closer to Syrian understandings of resilience:

In our Syrian culture, we don’t have Sumud, we have Sabr. Sabr means that I am patient for this thing that will happen. Sumud might mean strength for you; for us, we have it sabr. (Nour, Participant 2)

While “patience” in English sounds passive, Sabr is proactive. It is an active state of endurance as they await a purpose that transcends their current reality. Nadine reflected, “[NGO workers] did not affect me as much since I have Sabr…because I know these will become memories and we will continue a better life there, Inshallah.” These Arabic expressions reflected how resilience is narrated, sustained, and made culturally intelligible (see Table 2 for a summary of these expressions and their narrative functions).

**Table 2.** Arabic Expressions used by participants and their narrative functions.

Narrative function	Arabic script	Pronunciation	Literal translation	Typical use	Contextual meaning
*Spiritual Sumud*	إن شاء الله الفرج القريب	Inshallah al-faraj al-qarīb	God willing, relief will come soon	Spiritual reassurance in hardships	Hope for imminent relief
	رح يقدرنا الرب	Rah yiʾdarna al-Rab	God will appreciate us	Assurance sacrifice will be rewarded	Spiritual reward during fasting
	دعوة	Daawa	Prayer	Asking for or giving blessings	Camp community support through prayer
	الله يكثر خير	Allah ykater kheir	May God increase the good of…	Expressing thanks for support	Gratitude toward workers and authorities
	رحمة في قلبهم	Rahma fi qalbahum	Mercy in their hearts	Wishing others to act kindly	Prayers for compassion from authorities
	ما تقلقي، الله يفرجها	Ma teqlaqi, Allah yefrejha	Don’t worry, God will bring relief	Comforting reassurance	Reassuring friends/family members
Expressions of Suffering	ظلم	Tholm	Oppression/injustice	Describing acts of injustice	Talking about unfair treatment in life or war
	انقطع نسلنا	Nqataa naslana	It’s like our bloodline has ended	Extreme loss, generational fear	Describing trauma from war
	نحنا اتظلمنا	Nehna nthalamna	We have been wronged	Collective sense of injustice	Describing living circumstances for Arab women
	ّانكتب علي	Inkatab aalayna	It is written for me	Fate or destiny	Acceptance of struggles as preordained
	روحنا بكفنا	Rouhna bkafna	Our souls were in our hands	Fear from extreme danger	Emotional wounds of the sea
	ضاقت فينا الحياة	Daqet fina al-hayat	Life has closed in on us	Feeling trapped or hopeless	Feeling hopeless due to repetitive struggles
	غصة فينا	Ghasa fina	A lump in our throat	Emotional burden and exhaustion	Speaking about loss of past opportunities
	هم أكبر من همهم	Ham akbar mn hamhum	Burden bigger than theirs	Having greater struggles than others	Children holding burden bigger than them
Expressions of strength	لو قد ما الحياة أخذتك وجابتك بتضل واقف	Law ad ma al-hayat akhadetak w jebetak btdal waqef	No matter how much life takes you and brings you back, you stay standing	Self-motivation and resilience	Endurance through life’s hardships
	سند	Sanad	Support/pillar	Someone to lean on	Describing emotional and social support
	البنت الجسورة	Al-benet al-jasura	The brave girl	A self-image of feminine strength	Self-identification with resilience
	مكافحة	Mukafaha	Fighter	One who struggles and persists	Describing self-endurance
	رح ينتصر الحق	Rah yontosir Al-haq	Justice will prevail	Expressing faith in fairness	Hope in moral and social justice in war

*Note. *Expressions are grouped by narrative function, with transliterations reflecting participants' Levantine Syrian dialect. Literal translations give the word-for-word meaning, while contextual meanings show how participants used each expression during the interviews.

#### Subtheme 1.3. Fractured womanhood and reclaiming autonomy

War and displacement were only part of what shaped the women’s paths. In recounting personal stories, three women spoke not of war or borders, but of marriage and womanhood. They chose stories of raising children young, relinquishing dreams, and surviving restrictive expectations. Sarah became tearful when describing her Sumud story, “My marriage… I didn’t want to get married. I wanted to continue my education.” For many, Sumud meant enduring a path they did not choose, but imposed on them by societal pressure. Yet, it fueled their determination to break this cycle for their children. Nadine, for instance, left her town out of fear that her daughter would be forced to marry young, “There are political groups, they control the women there. I was scared. I took my daughter and came here.”

Layla described fighting for education and her desire to reclaim interrupted dreams after receiving her asylum. She is interested in finishing her degree and learning to drive. Her goal, she noted, was not to reject culture or family; but rather choose her own path. These struggles were a shared experience among Syrian women in the camp:

We [female friends] had a conversation about how much we’ve endured…We were like a few souls, and one woman was driving it…Inshallah, we’ll get the chance to learn. (Layla, Participant 1)

Zahra, often described as the mother of her group, added, “Sometimes we get depressed, we sit and cry together…I give them Sabr…This is how we strengthen each other. One collapses then another stands.” These matching wounds created a connection of support and shared hope for a better future.

### Theme 2. “Roots without soil”: The silent weight and wounded grace of motherhood in exile

To speak of themselves was to speak of motherhood; the two identities were woven together too tightly to ever stand apart. Their expression of Sumud; their purpose, motivation, sorrows, and sacrifices were inseparable from their children and families. This theme explores the complexities of motherhood in displacement.

#### Subtheme 2.1. Solo motherhood and the weight of maternal guilt

Motherhood for the women was not static, but continually reshaped by exile. Seven women became solo mothers either through death, separation, or husbands awaiting reunification. This transformation meant a shift in their parental role, assuming full emotional and physical responsibility of parenting:

The mother, the word mother is enough for all that [Sumud]. My children are in their teenage years and they tire of me, and their father is not here. This is enough because I have Sumud for them. (Nadine, Participant 4)

Maternal guilt was a recurring emotion. Nour, for example, left two of her children behind with her parents as she crossed the sea and awaits asylum, “I left them, but I left a piece of my heart there.” Her sacrifice, though understood by her as Sumud, was met with judgment from others in the camp saying, “What kind of mother leaves her children?” Alongside the guilt and social shame, Nour faced bureaucratic threats over her parenting. She explained, “When I first came here my daughter went missing, so the police threatened that if I lost her again, they would take her from me.” Parenting under social and structural surveillance has led to hyperattachment between mothers and children. Amal shared a similar dynamic, “They [children] never leave me, even now for the interview I had to convince them to let me come. They can’t go on without me.”

#### Subtheme 2.2. Mirroring strength and transgenerational Sumud

When asked about Sumud, the women’s first thought was their children, the living reason and reflection of their endurance. Nour shared, “I think of my children first. I need to have Sumud so I can stay strong for them and for myself; so that they can live a life that is better than this one.” Zahra mirrored Nour, “They strengthen me when I see the happiness and success in their eyes.”

A reciprocal process emerged: mothers and children mirror each other’s strengths and vulnerabilities. Nour recalled constantly crying when she first arrived. As time went by, she realized her children cried with her:

So I told myself that I shouldn't be like this. From your weakness, your children will also be weak…I need to be strong in front of my children. (Nour, Participant 2)

The psychological wounds of sea crossing were particularly visible in children. This did not go unnoticed by the mothers. Layla’s children were not aware of the crossing, they thought it was a trip, until they started drowning, “…They started holding me and saying, we don’t want anymore, take us back home. Now, even going to the beach, they get fearful.” Mothers spoke of the desire to restore childhoods disrupted by war and displacement. Layla hoped her children would, “see themselves just like the other children and forget the days of war and injustice (tholom).”

To shield their children from trauma, mothers rarely spoke directly about the journey. Instead, they reframed the experience by constructing alternative narratives of bravery and hope. Layla told her son he was a hero for crossing the sea, “I told him you are a hero. You were able to pass the sea and the mountains.” Different strategies were used by the women to reinforce Sumud within their children. They used imaginative, spiritual, or future-oriented strategies. In one case, Farah, whose child is still an infant, wishes to build Sumud through storytelling:

When she grows up, I’ll tell her about what we went through; about our people, about my life, and what happened to us. I’ll tell her how we became stronger, how people changed because they achieved their goals. (Farah, Participant 6)

**Figure 3.**
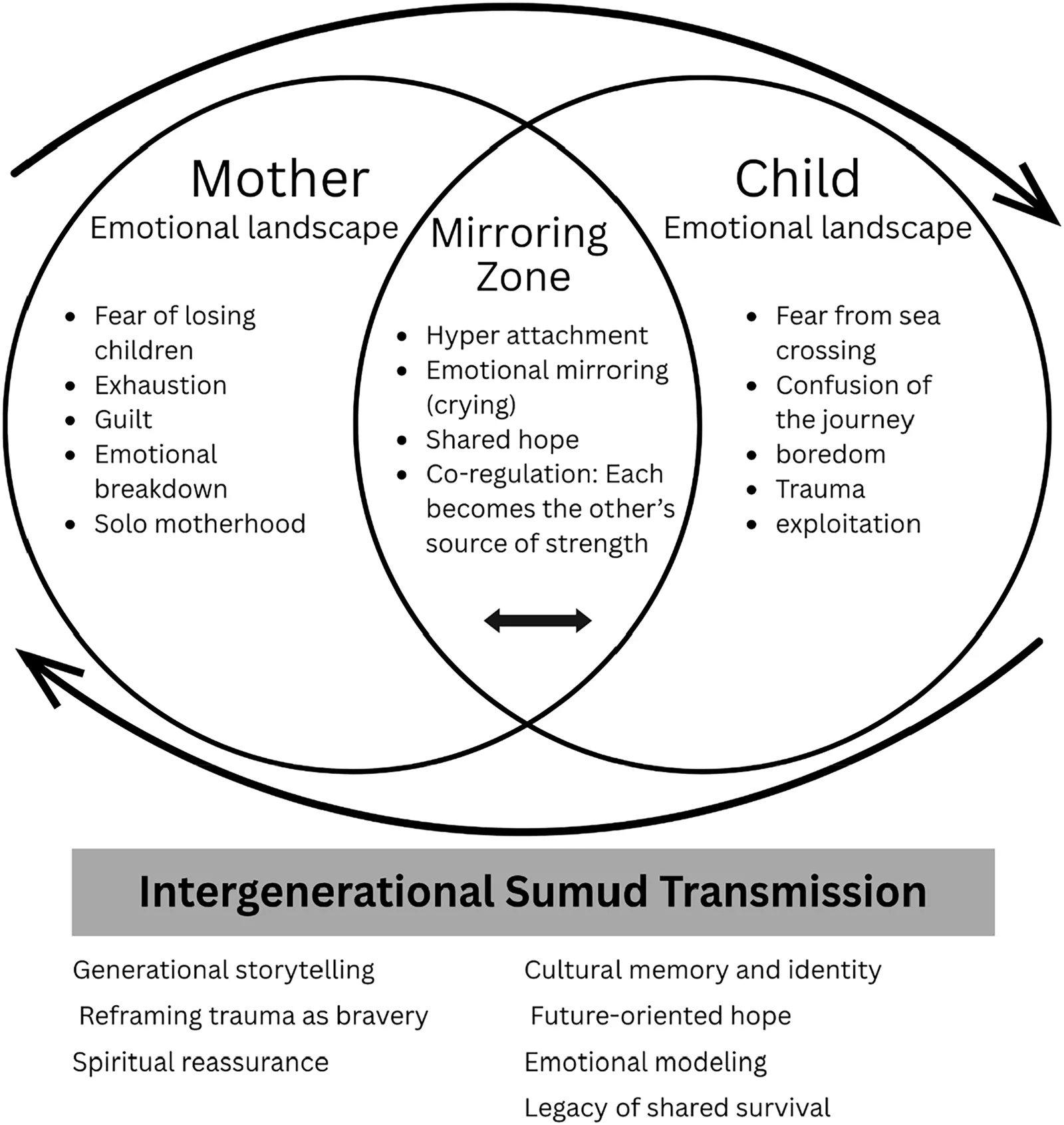
Conceptual framework for Mothers’ and children’s resilience. *Note*. Conceptual framework for Mothers’ and children’s resilience and the intergenerational transmission of Sumud.

Layla, on the other hand, told her children that their deceased father was watching over them. She offered spiritual reassurance and emotional continuity as a form of Sumud. The dynamics of this intergenerational transmission of Sumud between mother and child are illustrated in Figure 3.

### Theme 3. The Paradox of Sanctuary and life in limbo

**Figure 4.**
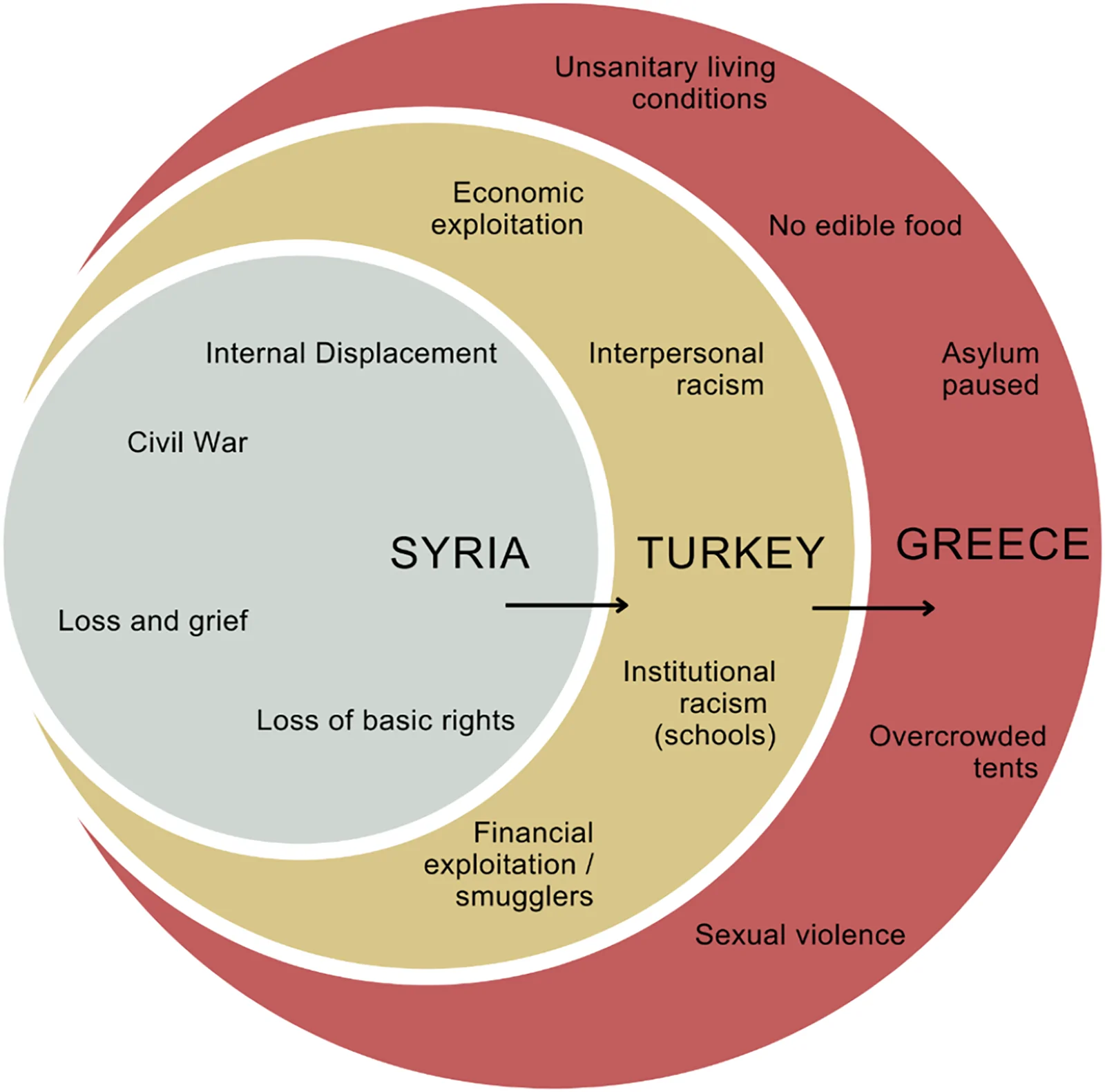
A layered visualization illustrating the overlapping hardships encountered across the Syria -Turkey - Greece Journey.

Sanctuary in the context of forced displacement rarely offers the peace it promises. While reaching safety is a necessity, arrival to the host country introduces new layers of difficulty. For the women, sanctuary is not experienced as resolution but as suspension. In this theme, the analysis focuses on the journey taken by the women and children from Syria, across Turkey, to Lesvos camp (see Figure 4 for the layered hardships encountered across this route).

#### Subtheme 3.1. Harrowing journey to safety

All eight women traveled the same path; fleeing Syria, passing through Turkey, and crossing the Aegean sea into Lesvos. For some, displacement began before leaving Syria. Layla recalled being internally displaced while receiving aid from a mosque, describing it as collective collapse and exhaustion. Zahra also voiced her frustration with the constant need to be strong, “We [Syrians] have Sumud like the mountain. We endured like the mountain, but more storms came until we collapsed.”

For those who resided in Turkey, different challenges emerged. All three women mentioned economic exploitation, racism, and constant fear for life. Sarah recalled how her husband, “Was very overworked and his health couldn’t handle it anymore”. Interpersonal racism became another pillar of emotional exhaustion. Nadine’s husband, for instance, was shot in the streets as he refused to give a Turkish man cigarettes and his phone.

Institutional racism was equally striking. Nour’s son faced segregation in the classroom, “My son used to go to school, they used to hit him. They used to put him in a room with other Syrians and the teacher did not teach them at all.” Nadine also recalled a teacher telling her children,“You are not supposed to be studying, you should go and work.”

Such dehumanization pushed many to risk the sea, which required access through smugglers who often exploited mothers and children traveling alone:

He [smuggler] used to take the young men and women and leave the mother and kids till the end. So if we were caught, the young ones would be able to flee. It’s like a trap… They don't care about the lives of the people. (Layla, Participant 1)

Crossing the sea was a sight of terror for mothers. Nadine wept remembering her children crying on the boat, “I felt guilty…when I saw them crying, I said I wish I didn’t take them.”

In these moments, Sumud fractured under the weight of guilt yet resurfaced in moments of need.

#### Subtheme 3.2. Life in the camp: Expectations versus realities

Arrival on the island shattered the women’s expectations of sanctuary. Layla described the camp as “a second war”:

I had high expectations for this place…but when we came here we were surprised. (Sarah, Participant 7)

Overcrowded tents, unsanitary conditions, and lack of adequate infrastructure defined daily life. The women feared their children’s safety. Farah mentioned placing napkins in her daughter’s ears as she slept in fear of cockroaches entering her ears. The lack of basic needs was a dominant topic, as distributed food was described by most as nonedible. Layla said she tries to swallow down the food for nutrition, but her children refuse to eat it, “It’s a shame…We bring it and throw it as is.”

The lack of basic needs was on the surface of a much deeper threat. Sexual violence, abuse, and harassment emerged as a horrifying reality. Amar shared the unspoken reality of rape and abuse that have never been admitted or recognized:

I personally know two people who died here, though no one admitted it was from rape or abuse. But we all know. There are girls walking around right now who are pregnant because of rape. (Amar, Participant 8)

Amar called out many unspoken issues, including the smuggling of drugs and alcohol inside the camp through children and the failure of camp security to provide safety and protection for women and children inside. Due to the camp’s overcrowding, families were forced to share the same tent. The lack of privacy has pushed families in communal living into a state of hypervigilance. Families in the same tent fight over simple things such as light preference, Layla explained. This tension left many in a state of social caution, causing many women to withdraw socially, “I don’t socialize a lot with others…there aren’t a lot of very good people.”

Amid the chaos, small pockets of solidarity still persisted. Community support systems appear to be a big factor in enduring camp life:

Life in the camp is both beautiful and miserable…because we started to feel like a family. It's like a family in exile. We sit, eat from one pot. I love that feeling. (Amar, Participant 8)

Nadine echoed, “We hang out with each other. We have parties…if a woman is leaving, we get happy for her. We do parties because it became a dream to be able to leave.” The sense of celebrating each other, strengthens their endurance and hope that they will share the same fate.

#### Subtheme 3.3. Stuck in limbo

As the asylum procedure for Syrians was paused in the EU, the fate of Syrian refugees became unknown. Hence, the wait has brought its own impact, Zahra explained, “We became very tired. We weren’t supposed to stay here for long. There is a lot of boredom here. We are not doing anything.”

Boredom and monotony emerged as central features of life in limbo. Days blurred into one another, producing frustration and division inside the camp. Layla compared it to every household scenario; “When you spend your day outside working, you will not have time to fight with your family…but if you are all day face to face, of course you will fight.”

Children felt the weight of limbo as well. Five participants mentioned their children saying “We are really bored, when will we leave?” Mothers felt unable to provide outlets of play, learning and connection. When asked about the way of strengthening Sumud in their children, most women said they encourage their children with promises of a better future. Nadine, like many others, reassured them with promises of reunion, “I tell them Inshallah we will go to your father and you will continue your life there. You will not stay here, you will continue your education.”

### Theme 4. NGO support and gaps in aiding Sumud

**Figure 5.**
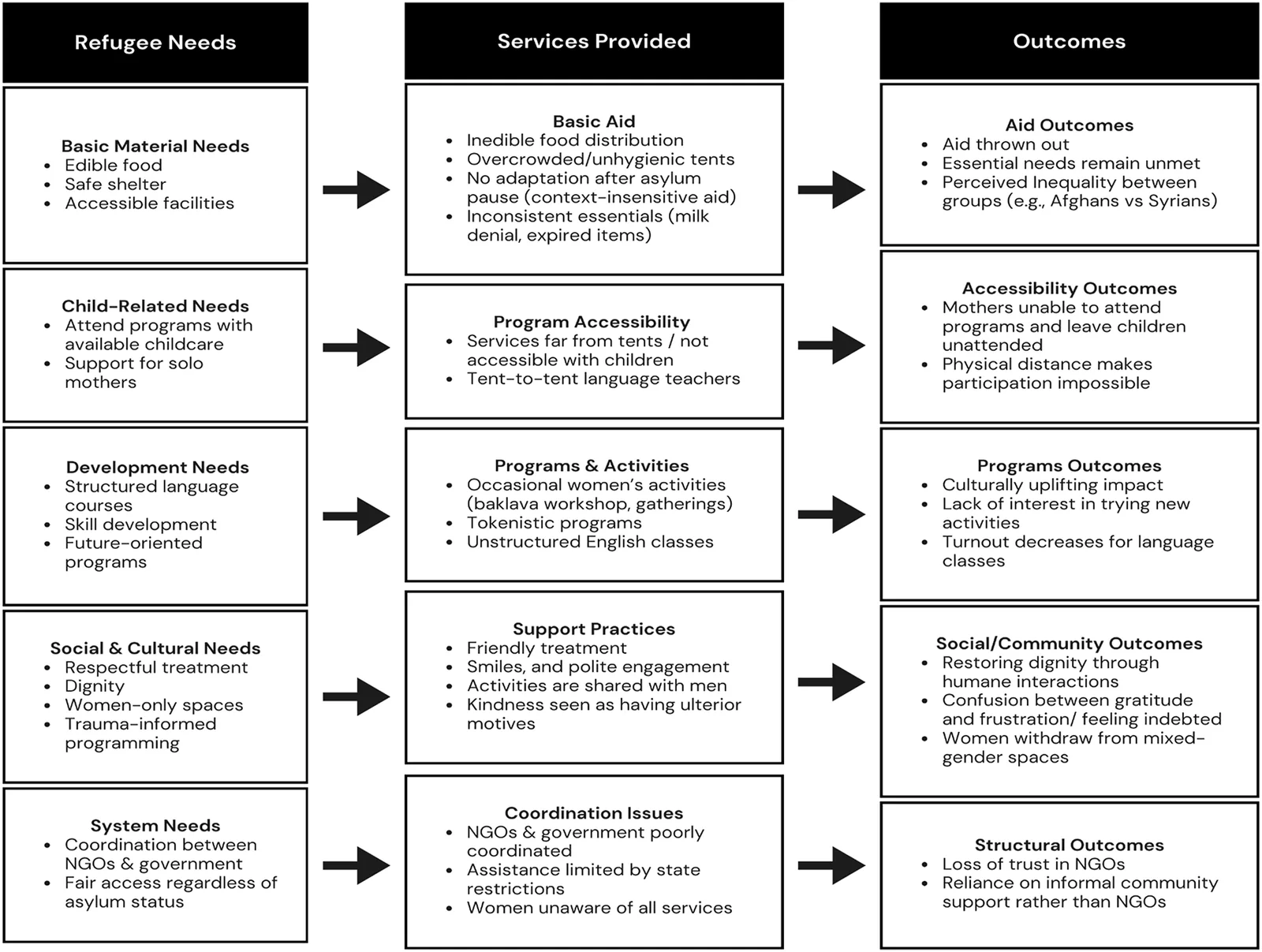
NGO support pathway. *Note*. NGO support pathway included refugee needs, services provided, and resulting outcomes.

The presence of NGOs within and around the refugee camp plays an integral part in the lives of Syrian women, making them the primary source of support and structured aid. This theme explores how Sumud is both supported and constrained by humanitarian structures, and how women’s perceptions shift as they engage more deeply with the humanitarian system (see Figure 5 for an overview of how refugee needs map onto services provided and resulting outcomes).

#### Subtheme 4.1. Dignity and the limits of kind treatment

Upon reaching the island, the kind treatment of NGO workers surprised most. Nadine shared, “We were deprived of that [respect] for a very long time…It is enough the smile that they give us, it makes us feel at ease.” Yet others, like Amar, long-term volunteer in two NGOs, had a different perception: “ Our [Syrians] resilience is about surviving…Theirs [NGOs]? using our pain to show off, “look how much we’re helping refugees” they show us off like trophies to raise donations. It’s not the same thing…it’s manipulation.”

#### Subtheme 4.2. The instrumentalization of suffering and asymmetries of power

Amar’s deep exposure to NGO’s internal systems brought attention to the unequal power dynamics and instrumentalization of refugee suffering. She also emphasized the nice treatment and invitation for tea gatherings is often used with an intention to convert the community’s religion. Amar’s statement evoked a contradiction; many women experienced support at the surface level such as friendly gestures, while they faced inaccessible or inconsistent assistance. Sarah, for example, praised NGOs’ treatments, but later expressed her frustration when she was denied milk for her child. “My infants were still babies, and they needed powdered milk… they kept refusing…The milk they give here at the camp is like blue water…No one drinks it.”

The women showed emotional confusion between cultural gratitude towards those who helped them and performative indebtedness. As Sarah put it, “As Syrians, we’re terrified of them [Europeans]. We do everything we can just to make them happy, just so they’ll accept us.” While gratitude remains central in Syrian culture, the women’s reflections exposed how dependency and inequality complicate genuine appreciation.

#### Subtheme 4.3. Tokenistic programming and unmet ecological needs

When discussing NGO programs, women described a range of services. Some mentioned support by distributing clothes, hygiene products, and other essentials. Others highlighted developmental activities, like language classes:

I registered [English class] and I went, but it's a normal class, not like a structured course. So I went twice or three times and I never went again. I didn't feel it was something serious. (Layla, Participant 1)

Five women mentioned learning language as their main interests in skill development. Yet, language classes were described as tokenistic support. Layla directed a message in her interview, “Give them [NGOs] the message to treat us like children. We go, we wouldn’t mind. From 8am till 1pm all day.” She explained that the classes are not tailored to their needs, as they are taught letters and colors, instead of daily conversations, “This is not what we want. We want to open up conversations.” These resources are requested to aid in navigating survival in their future host country. However, as the lack of structure increases, the interest of women decreases. Layla stated most of the women stopped attending after the first time.

Beyond tokenistic support, parental responsibility was another barrier limiting participation. Nour shared her interest in joining classes, “but I couldn’t leave my children because I was scared if they got lost or not to take care of them.” Nadine echoed her, “…Today I left her [toddler] asleep so that I can come do the laundry. I brought her siblings with me to help me.”

Many women also pointed to the lack of comprehensive programs tailored to their daily struggles. Basic resources such as laundry facilities are scattered inside and outside the camp and the lack of coordination between NGO and governmental support exacerbates the burden. Nadine, for example, is interested in improving her sewing skills, but she explained, “I spend the whole day grabbing food, cleaning products, cooking, and doing laundry in all different places…and the day ends so fast.” These logistical challenges inhibit women from participating in development-oriented programs.

While the asylum process was paused for Syrians, it was still ongoing for other refugees. Yet, some NGOs did not adjust their aid according to the changing circumstances:

There is this NGO for pregnant women and young children. When we go to them, they tell us you already got it and now it’s only for pregnant women…and they don’t negotiate. But the Afghans their asylum is resuming and leaving in two months. (Amal, Participant 3)

Still, several women benefited from certain women-specific initiatives. Layla and Sarah were excited in attending a Baklava-making workshop held during Ramadan. Sarah noted, “felt like a holiday.” Yet, many emphasized the need for more women-only spaces where they could relax and be themselves.

Some of the women’s requests were already being addressed, but they were unaware of it. Nadine, for example, expressed a desire to attend classes but cited childcare responsibilities as a barrier. Nour later noted that NGOs started sending language teachers tent-to-tent, tailoring programs for accessibility. Similarly, while Layla requested access to a hairdresser, Zahra mentioned that a small salon already exists where women provide such services. Several women were also unaware that the centers they visit are operated by NGOs, reflecting a gap in communication and outreach.

## Discussion

This study offers an in-depth exploration of how Syrian refugee mothers and children define, experience, and transmit resilience during their journey of displacement. The aim was not to evaluate resilience from a universal or western-driven lens, but rather center the women and children’s own narratives, language, practices, and interpretations. The findings reported here reflect the accounts of eight Syrian refugee mothers in one camp at one political moment. The interpretations offered are situated in that specific context and are not presented as generalizable claims about Syrian women as a whole. The research focused on three areas of exploration, (1) the lived experiences of Sumud across the present, past, and future, (2) the practice of passing on Sumud to the younger generation, and (3) the exploration of NGO’s alignment with Syrian’s culturally informed resilience.

### Resilience as Sabr: A culturally and linguistically grounded practice

A central finding of this study revealed that Syrian women in this study did not primarily describe their resilience through Sumud, but through Sabr. Sabr was mentioned repeatedly across interviews and surfaced as an emotional and spiritual backbone for the women as they faced ongoing adversities. While Sumud is a term with strong political, historical, and communal roots in Palestinian resistance literature, Sabr appeared as proactive endurance shaped through patience, persistence, and faith.^38^ Nour’s words even captured this determination, “Sumud might mean strength for you; for us [Syrians], we have it sabr.”

This linguistic and cultural emphasis contributes to resilience research in two ways. First, these findings support arguments that resilience cannot be translated uncritically across cultural and linguistic boundaries.^24,43^ This shows the importance of the native language and the expressions in the women’s accounts of survival. Second, it demonstrates that resilience cannot be solely explained in the language of “recovery” but can be understood as ongoing, fractured, and cyclical. Women narrated bombings, hunger, and displacement in the same breath as expressions of gratitude or hope such as Alhamdulillah and Inshallah. They also expressed moments of complete erasure of their strength, especially in instances of loss or separation from loved ones. This reflects resilience as a spectrum, fleeting and wavering with the fluctuation of external factors. This also echoes previous literature which argues that resilience in forced migration is not about recovery but about sustaining life under chronic adversity.^44^

This pattern carries a methodological lesson about the use of culturally borrowed idioms in resilience research. The study began with Sumud as its organizing concept, drawing on its rich theorization in Palestinian scholarship. The women, however, more readily named their experience as Sabr. This gap matters. Sumud and Sabr are not interchangeable. Sumud is rooted in a specific history of collective, place-based resistance. Sabr is grounded in faith and patient endurance and circulates widely across Arabic-speaking Muslim communities. Importing Sumud as a ready-made lens therefore risked framing the women’s resilience in terms that were not fully their own. The broader point is that culturally specific concepts travel unevenly across communities. What is analytically generative in one setting can obscure meaning in another. For this group of Syrian mothers, Sabr proved the more faithful organizing concept. Future research using culturally grounded idioms should treat such terms as open questions to be tested against participants’ own language, rather than as categories applied in advance. This does not discard Sumud. It repositions it as one resource among several, to be drawn on where it reflects how a community actually narrates its endurance.

### Resilience in limbo: Challenging the myth of sanctuary

A second key finding was the sharp contradiction between the expectation of “reaching safety” and the reality of life in Lesvos camp. Although arriving to the island signified survival from immediate dangers, it still ushered in a new phase of hardships. Greece was described as a “second war” with overcrowded tents, absent security, inadequate food, lack of privacy, and sexual violence. This resonates with the Life in Limbo framework where displacement is not only a past trauma to recover from but as an ongoing state of liminality, where futures remain indefinitely deferred.^37^ In the Greek asylum context, limbo is not only an emotional state but also a structural one. The pausing of Syrian asylum applications in early 2025 produced another layer of uncertainty intensifying fear, boredom, and stagnation. Women repeatedly mentioned boredom, stagnation, days blurred into each other, and their children asking “when will we leave?” This duality complicates the framing of “arrival” as an endpoint of suffering.^9^

These insights highlight how resilience cannot be meaningfully understood nor supported without acknowledging the structural conditions that constrain their agency. “Resilience” responsibility still falls on those who are displaced while the failure of authorities to provide safety, basic needs, and just protocols still persists. Their testimonies of stillness reveal that limbo itself is a form of structural violence which places the burden of coping on the community while overlooking the systems that create and maintain their vulnerability.^29–31^ The women were not “failing to be resilient”; they were surviving within conditions that systematically undermined the very possibility of stability.

### Autonomy, motherhood, and collective womanhood as anchors of resilience

A recurring theme revealed that resilience is not only cultural but also deeply gendered, bound to the intersecting role of womanhood, motherhood, and autonomy in exile. Nadine’s words captured this intersection, “The Syrian woman is very resilient, she endures a lot, she endures tiredness and thinking. She endures the absence of her husband, she endures losing her brother, sons, daughters, husband. She lives on her own, and she continues her journey on her own.” Motherhood played an integral role in the women’s definition of resilience. Women described their children as both the source and purpose of their resilience. Sumud and Sabr were not only described as personal endurance but as a maternal duty towards their children.

Beyond the mother–child dyad, women found strength in their friendships with each other. Resilience was nurtured and encouraged through strong bonds of shared womanhood creating “a family in exile,” as they share their common past and present experiences, while strengthening each other towards the future. The solidarity between the women in sharing Sabr, Sumud, and suffering reinforces the significance of communal resilience and collective forms of endurance among displaced Syrian women.^45^

These findings reinforce the evident presence of intersectionality. Resilience was not shaped by gender alone, but by the convergence of motherhood, cultural norms, and displacement. Women’s resilience was simultaneously demanded by patriarchal norms, constrained by camp conditions, and reinforced by womanhood and autonomy practices.^35^ These stories echo feminist migration scholarship, which frames displacement as loss and opportunity for renegotiating gendered constraints.^46^

### NGOs as supportive and constraining forces

The relationship between the community and NGOs was complex. Many women expressed gratitude towards the humane treatment and respect from NGO workers. Simple acts of smiling, shared meals, and respectful communication restored dignity that felt vanished after years of dehumanization. This dignified treatment in itself reinforced their sense of resilience. These same positive experiences also stood in sharp contrast with recurring gaps and frustrations. The overcrowded camp, deprivation, insecurity, and violence have caused the women to alternate between gratitude and despair towards both Greek authorities and NGOs.

Some statements of the women revealed failure of NGOs to meet foundational ecological needs. Participants described expired baby milk, overcrowded and unsafe housing, inedible food, lack of child support, dispersed aid, and poorly structured programs. These findings indicate that resilience-supportive programming must begin with ecological foundations such as safety, childcare, hygiene, food, and shelter before moving to psychosocial or developmental services.^19^ The gaps also showed structural failure in coordination between NGOs and governmental institutions in providing these needs. This clear gap between surface-level policy and real application on the ground deepens the community’s disillusionment. There is a significant need for community-led committees in addressing these gaps.^20^ The women’s requests were consistent, clear, and asserted the need for their integration in policymaking: structured language classes, women-only spaces, childcare support, practical integration skills, and culturally grounded programming. The gap between program design and women’s daily realities reveals how humanitarian structures often fail to consider the intersecting pressures of motherhood, culture, and limbo.

These observations return the discussion to the decolonial critique of resilience set out earlier. When essential needs such as edible food, safe shelter, childcare, and coordinated services go unmet, the language of resilience can quietly shift responsibility onto the women themselves. Praising the endurance of mothers while leaving the conditions of the camp unchanged individualizes what is in fact structural precarity. The women’s Sabr should therefore be read as a measure of the burden placed on them, and not as evidence that the system is working. This reading does not require discarding existing humanitarian frameworks. Sabr and Sumud can be understood as culturally specific expressions of the faith-sensitive and culturally grounded resilience already named in humanitarian practice (Figure 1). The contribution here is to show what such frameworks look like once they are filled in with a community’s own vocabulary and priorities. The concern is with how dominant resilience discourse is applied, in ways that treat endurance as an individual achievement and overlook the institutional conditions that make endurance necessary. Read this way, the findings extend faith-sensitive frameworks from within, while holding onto the decolonial point that resilience must not become a substitute for structural responsibility.

### Recommendations and implications for practice

The findings of this study have important implications for humanitarian practice. First, the findings call for a reorientation of NGO interventions. Programs for Syrian mothers and children must be designed to fit the community’s definition and practice of resilience. That involves recognition of their Sabr, religious faith, importance of gender-specific practices, and maternal responsibilities they hold. This involves employing more Arabic-speaking, culturally competent women volunteers who are capable of grasping the needs and recommendations of the community.

Second, interventions must also prioritize ecological foundations such as safety, shelter, edible food, legal aid, and childcare. These essential aids do not often require more financial assistance but rather redirection of current assistance. For example, as requested by some women, providing nonedible meals that are thrown out by the community can be replaced by a small monthly stipend for each family. Strengthening coordination between NGOs and state authorities is also necessary to reduce fragmentation in the delivery of essential services, which currently forces women to spend much of their day navigating scattered resources.

Third, the findings underscore the urgent need for professionalization within humanitarian roles that directly affect women’s daily functioning. Women in this study described inconsistency, poor structuring of programs, and cultural mismatch, which are often linked to the fact that many frontline services are implemented by short-term volunteers rather than trained professionals.^11^ The high turnover of volunteers, combined with the lack of rigorous preparation in psychosocial or educational programming, contributes directly to the gaps identified. Volunteers should receive extensive training before engaging in psychosocial, educational, or resilience-building activities.

Fourth, gender-sensitive design must become standard practice. Women repeatedly emphasized the need for women-only spaces whether for classes, celebrations, mental health groups, or informal gatherings where they could participate freely without fears of surveillance, shame, or cultural discomfort. Programs should also account for maternal responsibilities by offering flexible scheduling, childcare options, and safe spaces for children during activities.

Finally, in order for NGOs to move away from tokenistic programs, there is a vital need for participatory, community-led committees that engage women as partners rather than passive recipients. NGOs should recognize the importance of dignity, equality, and respect in everyday interactions as resilience-supportive practices. Humane treatment by staff, women’s access to collective spaces, and opportunities for education and self-reliance can foster resilience in ways that align with women’s own aspirations.

### Strengths and limitations

A central strength of this research lies in its focus on Syrian women’s own language and voice. Conducting interviews in Arabic allowed the participants to express themselves through cultural idioms that heavily resonate with them. Secondly, the study contributes insights into both the relational and structural dimensions of resilience, particularly the interplay between maternal duty, children’s agency, and NGO interventions which is rarely addressed in refugee literature.

This study also carries certain limitations. First, the sample size was small and limited to Syrian mothers in Lesvos, which restricts generalizability. Men’s perspectives, children’s direct voices, and experiences from other host contexts were excluded. Second, the study was conducted during a specific political moment when asylum processing for Syrians was suspended in Greece, which might have intensified the participants’ sense of limitation. Third, while reflexivity was practiced, researchers' role as both insider (Arabic-speaking volunteer) and outsider (academics) was both an advantage as well as a limitation as it influenced the data.

### Future research

Building on these limitations, this study opens several avenues for future research to emerge. Further work is needed to examine Syrian children’s culturally informed resilience. Studies involving art, storytelling, and child-centered methodologies could generate deeper insights into how children define, experience, and practice resilience. Comparative research should also explore the prominence of Sabr within the Syrian community, since more research is required to identify its relevance across different Syrian communities, across genders, as well as in different host countries. In addition, the study identifies a substantial gap between theoretical frameworks of NGO programming and its application on the ground.

## Conclusion

This study shows how Syrian refugee mothers on Lesvos sustain themselves and their children through culturally grounded, relational, and spiritual practices of resilience. Their daily acts of Sabr (patient, proactive endurance) reveal resilience not as an individual trait or linear recovery, but as a culturally embedded practice intertwined with motherhood, decision making, and the navigation of fragile futures. Motherhood emerged as a central anchor of emotional and moral strength, while children contributed actively to their mothers’ endurance, illustrating resilience as intergenerational and reciprocal even in conditions of profound instability.

The women’s narratives also challenge humanitarian assumptions that arrival in Europe constitutes safety. The suspension of Syrian asylum applications and deteriorating living conditions created structural limbo that undermined their agency and well-being, despite moments of dignity restored through humane NGO interactions. Their accounts expose a persistent gap between policy intentions and lived realities, underscoring the need for humanitarian approaches that move beyond universal models toward culturally grounded, co-designed interventions. Resilience cannot be placed solely on women and children; it requires structural responsibility to ensure safety, dignity, and viable futures for displaced communities.

## Supplemental material

Supplemental material - *“I tell them to have Sumud, have Sabr, for your children, for yourself”*: Culturally informed resilience and humanitarian gaps among Syrian refugee women in Greece Supplemental material for *“I tell them to have Sumud, have Sabr, for your children, for yourself”*: Culturally informed resilience and humanitarian gaps among Syrian refugee women in Greece by Karen Youssef, Gulnaz Anjum, Halina Grzymała-Moszczyńska, PhD in Women's Health

## Data Availability

We have provided supplemental information and additional details for this paper along with the submission. Additional anonymized data will be provided upon request. Please reach out to the corresponding author.*
