# Supplementary material for: “I tell them to have Sumud, have Sabr, for your children, for yourself”: Culturally informed resilience and humanitarian gaps among Syrian refugee women in Greece
Source: Womens Health (Lond). 2026 Aug 20;22:17455057261473454. doi: 10.1177/17455057261473454 (PMC13494308; doi:10.1177/17455057261473454)
Supplement: Supplementary file 1 [file sj-pdf-1-whe-10.1177_17455057261473454.pdf]

Appendix A - Information Letter and Consent Form (English and Arabic)

Are you interested in taking part in the research project

"**Resilience but for whom?": A Phenomenological Study on Western-Driven Interventions and Its Detachment from Culturally-Informed Resilience "Sumud" Within Syrian Refugees in Greece**"

Purpose of the project

You are invited to participate in a research project where the main purpose is to explore the culturally-informed resilience, "sumud", within Syrian refugee women and children in Mavrovouni camp in Lesvos, Greece. Through semi-structured interviews with mothers and psychosocial activities with children, this study aims to understand their own definition of resilience, its connection to their past, present, and future, how they pass it on to younger generations, and its alignment with NGO interventions in fostering resiliency.

The interview will be conducted with Arabic speaking and self-identified Syrian mothers who are current refugees in Lesvos island.

The anonymized interviews will be analyzed and discussed in a masters thesis and may subsequently be used for presentations, academic and popular science publications where your identity will continue to be unidentifiable.

Which institution is responsible for the research project?

University of Oslo will be responsible for this Master's research project. Project coordinators are main supervisor Professor Halina Grzymała-Moszczyńska, Associate Professor Gulnaz Anjum, and Global Minds master student Karen Youssef.

Why are you being asked to participate?

You are being contacted because this study aims to explore the culturally-informed resilience, "sumud", within Syrian mothers that are older than 18 years old, have children between the ages 6-15, and are currently refugees living in Mavrovouni camp in Lesvos, Greece.

What does participation involve for you?

Individual interviews of approximately 1 hour will be conducted, during which audio recordings and notes of the interview will be taken. This information will only be shared with the master's student and the supervisor.

Participation is voluntary

Participation in the project is voluntary. If you chose to participate, you can withdraw your consent at any time without giving a reason. All information about you will then be made anonymous. There will be no negative consequences for you if you choose not to participate or later decide to withdraw. However, after data is anonymised, it will not be possible to remove your information since it will be unable to identify which information came from you.

Your personal privacy– how we will store and use your personal data

We will only use your information for the purposes we have explained in this letter. We treat the information confidentially and in accordance with privacy regulations. The audio recordings will be stored electronically on a secure platform for UiO, Services for sensitive data, TSD. Audio recordings will be deleted once all interviews have been transcribed. The identifiable information such as your name, study/workplace, position will be stored separately from audio recordings and will be deleted after the research period set for October 1st, 2025. Participants of the project will not be able to be identified or recognized in the publication of the thesis. Total anonymised transcribed data will be stored internally and may be reused for research purposes.

What gives us the right to process your personal data?

We process your information for research purposes in the public interest.
Based on an agreement with University of Oslo, The Data Protection Services of Sikt Norwegian Agency for Shared Services in Education and Research has assessed that the processing of personal data in this project meets requirements in data protection legislation.

Your rights

So long as you can be identified in the collected data, you have the right to:

- access the personal data that is being processed about you
- request that your personal data is deleted
- request that incorrect personal data about you is corrected/rectified
- receive a copy of your personal data (data portability), and
- send a complaint to the Norwegian Data Protection Authority regarding the processing of your personal data

Where can I find out more?

If you have questions about the project, or want to exercise your rights, contact:

Project coordinator Halina Grzymała-Moszczyńska at SWPS

- halina.grzymala-moszczynska@ignatianum.edu.pl

Project coordinator Gulnaz Anjum at UIO

- gulnaz.anjum@psykologi.uio.no

UiO's Data Protection Officer Roger Markgraf-Bye

- personvernombud@uio.no

Project responsible student Karen Youssef

- Kareny@uio.no

If you have questions about how data protection has been assessed in this project by Sikt, contact:

- email: personverntjenester@sikt.no or by telephone: +47 73 98 40 40.

Yours sincerely,

Halina Grzymała-Moszczyńska	Karen Youssef
(Project supervisor)	(project responsible student)

Declaration of Consent

I____

have received and understood information about the project: Qualitative research on “Resilience but for whom?”: A Phenomenological Study on Western-Driven Interventions and Its Detachment from Culturally-Informed Resilience “Sumud” Within Syrian Refugees in Greece”, and have had the opportunity to ask questions. I agree:

● to participate in an interview with audio recordings and notes
● that audio recordings, notes and personal data are stored until the end of the project (01.10.2025)

I consent to my data being processed until the project is completed

Signature _____________________________

Date _____________________________

هل أنتِ مهتمة بالمشاركة في مشروع البحث:

الصمود، ولكن لمن؟ : دراسة ظواهرية حول الأنشطة الغربية وانفصالها عن مفهوم"الصمود " الثقافي لدى اللاجئين السوريين في اليونان

هدف المشروع

أنتِ مدعوة للمشاركة في مشروع بحثي يهدف إلى استكشاف مفهوم "الصمود" كمرونة ثقافية لدى النساء والأطفال اللاجئين السوريين في مخيم "مفروفوني" في جزيرة ليسبوس باليونان. من خلال مقابلات شبه منظمة مع الأمهات وأنشطة نفسية اجتماعية مع الأطفال، يسعى هذا البحث إلى فهم تعريفكِ الشخصي للصمود، علاقتها بماضيكِ وحاضركِ ومستقبلكِ، وكيفية نقلها للأجيال القادمة، ومدى توافقها مع تدخلات المنظمات غير الحكومية في تعزيزها.

ستُجرى المقابلات مع نساء سوريات يتحدثن العربية ويعرفن أنفسهن كأمهات لاجئات يقمن حالياً في جزيرة ليسبوس. سيتم تحليل المقابلات بشكل مجهول وستُناقش في أطروحة ماجستير، وقد تُستخدم لاحقاً في عروض تقديمية أو منشورات أكاديمية وشعبية مع ضمان عدم الكشف عن هويتكِ.

أي مؤسسة مسؤولة عن مشروع البحث؟

جامعة أوسلو هي المسؤولة عن مشروع البحث الخاص بأطروحة الماجستير. ومنسقو المشروع هم الأستاذة المشرفة الرئيسية هالينا غرزيمالا-موسزتشينسكا,الأستاذة المساعدة غولناز أنجم ,طالبة الماجستير في برنامج Global Minds كارين يوسف

لماذا طُلب منكِ المشاركة؟

تم الاتصال بكِ لأن هذه الدراسة تهدف إلى استكشاف مفهوم "الصمود" كمرونة ثقافية لدى الأمهات السوريات اللاجئات ممن تجاوزن سن 18 عاماً ولديهن أطفال تتراوح أعمارهم بين 6 و15 عاماً، ويعشن حالياً في مخيم "مفروفوني" بجزيرة ليسبوس.

ماذا تتطلب المشاركة منكِ؟

ستُجرى مقابلات فردية تستغرق حوالي ساعة واحدة، يتم خلالها تسجيل الصوت وتدوين الملاحظات. هذه المعلومات ستُشارك فقط مع طالبة الماجستير والمشرفة.

المشاركة طوعية

المشاركة في المشروع اختيارية تماماً. إذا اخترتِ المشاركة، يمكنكِ سحب موافقتكِ في أي وقت دون الحاجة لتقديم سبب. في حال انسحابكِ، سيتم جعل جميع المعلومات عنكِ مجهولة. لن تكون هناك أي عواقب سلبية إذا اخترتِ عدم المشاركة أو قررتِ الانسحاب لاحقاً. ومع ذلك، بعد جعل البيانات مجهولة، لن يكون من الممكن حذف معلوماتكِ لأنها لن تكون قابلة للتحديد.

خصوصيتكِ الشخصية – كيف سنحفظ بياناتكِ الشخصية ونستخدمها؟

- سنستخدم معلوماتكِ فقط للأغراض التي شرحناها في هذه الرسالة. ونتعامل مع المعلومات بسرية ووفقاً للوائح الخصوصية
- سيتم تخزين تسجيلات الصوت إلكترونياً على منصة آمنة خاصة بجامعة أوسلو (خدمات البيانات الحساسة – TSD)
- سيتم حذف التسجيلات الصوتية بعد تفريغ جميع المقابلات.
- لمعلومات القابلة للتعريف، مثل اسمكِ أو مكان دراستكِ/عملكِ أو منصبكِ، ستُخزن بشكل منفصل عن التسجيلات الصوتية وستُحذف بعد انتهاء فترة البحث في 1 أكتوبر 2025
- لن يتم التعرف على المشاركات في المشروع أو تحديدها عند نشر الأطروحة.

ما الذي يمنحنا الحق في معالجة بياناتكِ الشخصية؟

نقوم بمعالجة معلوماتك لأغراض بحثية من أجل المصلحة العامة.
بناءً على اتفاق مع جامعة أوسلو، قامت خدمات حماية البيانات التابعة لوكالة "SIKT" النرويجية بتقييم أن معالجة البيانات الشخصية في هذا المشروع تتوافق مع متطلبات تشريعات حماية البيانات.

حقوقكِ

طالما يمكن تحديد هويتكِ في البيانات التي تم جمعها، لديكِ الحق في
- الوصول إلى البيانات الشخصية التي تتم معالجتها عنك
- طلب حذف بياناتكِ الشخصية
- طلب تصحيح/تعديل أي بيانات شخصية غير صحيحة
- الحصول على نسخة من بياناتكِ الشخصية (قابلية نقل البيانات)
- تقديم شكوى إلى هيئة حماية البيانات النرويجية بشأن معالجة بياناتكِ الشخصية

إذا كان لديكِ أي أسئلة أو استفسارات، لا تترددي في التواصل

- منسقة المشروع هالينا غرزيمالا-موسزتشينسكا في SWPS
halina.grzymala-moszczynska@ignatianum.edu.pl

- منسقة المشروع جولناز أنجوم في UIO
gulnaz.anjum@psykologi.uio.no

- مسؤول حماية البيانات في UiO، روجر ماركغراف-باي
personvernombud@uio.no

- الطالبة المسؤولة عن المشروع كارين يوسف

kareny@uio.no

إذا كانت لديك أسئلة حول كيفية تقييم حماية البيانات في هذا المشروع بواسطة Sikt, فاتصل بـ:

البريد الإلكتروني: personverntjenester@sikt.no أو عبر الهاتف: +47 73 98 40 40.

مع كامل التقدير

هالينا جرزيمالا-موسزكزينسكا
(مشرف المشروع)

كارن يوسف
(الطالب المسؤول عن المشروع)

إقرار بالموافقة

أنا ____

قد تلقيت وفهمت المعلومات المتعلقة بالمشروع

الصمود، ولكن لمن؟ : دراسة ظواهرية حول الأنشطة الغربية وانفصالها عن مفهوم"الصمود " الثقافي لدى اللاجئين السوريين في اليونان

وقد أتيحت لي الفرصة لطرح الأسئلة. أوافق على

- المشاركة في مقابلة مع تسجيلات صوتية وتدوين ملاحظات
- تخزين التسجيلات الصوتية و الملاحظات والبيانات الشخصية حتى نهاية المشروع (01.10.2025)

أوافق على معالجة بياناتي حتى اكتمال المشروع.

التوقيع: ______________________________

التاريخ: ______________________________

Appendix B - Interview Guide (English and Arabic)

Interview Guide

Background information

1. Can you tell me about yourself
    - Your name
    - Age
    - Where are you from originally
    - What is your family status
    - How many kids do you have, how old are they

Journey

2. Can you tell me about your journey to Lesvos
    - Where did you come from and how long have you been on the island
    - Who did you come with
3. What made you decide to seek asylum in Lesvos?
4. How would you describe your journey from your homeland up until arriving to Lesvos (emotions, challenges, struggles)

Life in the camp

5. How would you describe your daily life in the camp right now
    - Describe the living situation
    - How do you spend your time (inside and outside of the camp)
6. How is the community in the camp (the relationship with other refugees and local people)

Sumud

Sumud Meaning as an Abstract Thought

7. What does the word “sumud” mean to you? Who/what do you think of?
8. What does sumud mean in syrian culture and for syrian refugees particularly

Sumud in Practice

9. How does the word “sumud” fit in your journey coming here
10. How do you feel you practice sumud in your daily life here in lesvos
11. How do you think “sumud” will link to your future journey after leaving the island
12. Can you tell me a story of a situation in which you practiced sumud in the face of adversity? (provide examples)
    - where /when/ what situations or people who make you feel Sumud the most?
    - Can you describe the emotions that you feel are accompanied with Sumud?

Transgenerational Sumud

Sumud in Children

13. Do you try to share this concept of sumud with your children, if so how?
14. How do you see/view sumud within your children?
15. What are the factors that you think strengthens sumud within your children?

NGO interventions and Sumud

16. Can you describe any experiences you’ve had with NGOs during your journey?
17. What kind of help did you expect to receive on the island before arriving here?
18. How do you feel about their role in supporting resilience?
19. What do resilience and *sumud* mean to you, and do you see any connections or differences in how NGOs approach them?
20. What do you think would make NGO support more meaningful for you and your resilience through this journey?

دليل المقابلة

المعلومات الأساسية

1. هل يمكنك أن تخبريني عن نفسك؟
   - اسمك
   - عمرك
   - من أين أنتِ أصلاً؟
   - ما هي حالتك العائلية؟
   - كم لديكِ من الأطفال؟ وما أعمارهم؟

الرحلة

2. هل يمكنكِ أن تخبريني عن رحلتكِ إلى ليسفوس؟
   - من أين أتيتِ وكم مضى عليكِ على الجزيرة؟
   - مع من وصلْتِ إلى الجزيرة؟
3. ما الذي دفعكِ لاتخاذ قرار طلب اللجوء في ليسبوس؟
4. كيف تصفين رحلتكِ من وطنكِ الأم إلى وصولكِ إلى ليسفوس؟ (المشاعر، التحديات، الصعوبات)

الحياة في المخيم

5. كيف تصفين حياتكِ اليومية في المخيم حالياً؟
   - صفي وضع المعيشة في المخيم.
   - كيف تقضين وقتكِ داخل المخيم وخارجه؟
6. كيف هو حال المجتمع في المخيم؟ (علاقتكِ مع اللاجئين الآخرين والسكان المحليين)

الصمود

الصمود كفكرة مجردة

7. ماذا تعني لكِ كلمة "صمود"؟ من أو ما الذي تفكرين فيه عند سماع الكلمة؟
8. ما معنى "الصمود" في الثقافة السورية وبالنسبة للاجئين السوريين بشكل خاص؟

الصمود في الممارسة

9. كيف تجدين أن كلمة "صمود" تتناسب مع رحلتكِ للوصول إلى هنا؟
10. كيف تمارسين الصمود في حياتكِ اليومية هنا في ليسفوس؟
11. كيف تعتقدين أن "الصمود" سيكون مرتبطاً برحلتكِ المستقبلية بعد مغادرة الجزيرة؟

12. هل يمكنكِ أن تخبريني عن موقف أو قصة واجهتِ فيها صعوبة كبيرة ومارستِ فيها الصمود؟ (قدّم أمثلة)

- متى وأين ومع من تشعرين بالصمود أكثر؟
- هل يمكنكِ وصف المشاعر التي تصاحب إحساسكِ بالصمود؟

الصمود عبر الأجيال

الصمود عند الأطفال

13. هل تشاركين مفهوم "الصمود" مع أطفالكِ؟ إذا كان كذلك، كيف؟
14. كيف ترين "الصمود" في أطفالكِ؟
15. ما هي العوامل التي تعتقدين أنها تقوي "الصمود" لدى أطفالكِ؟

تدخلات المنظمات غير الحكومية والصمود

16. هل يمكنك وصف أي تجارب مررت بها مع المنظمات غير الحكومية خلال رحلتك؟
17. ما نوع المساعدة التي كنت تتوقع الحصول عليها في الجزيرة؟
18. كيف تشعر تجاه دورها في دعم الصمود؟
19. ماذا تعني لك المرونة و"الصمود"، وهل ترى أي تشابه أو اختلاف في كيفية تعامل المنظمات غير الحكومية معهما؟
20. برأيك، ما الذي يمكن أن يجعل دعم المنظمات غير الحكومية أكثر معنى بالنسبة لك

Appendix C - UiO Research Ethics Committee Approval

UiO : University of Oslo
Faculty of Social Sciences – Departement of Psychology

Karen Youssef
Halina Grzymała-Moszczyńska
Gulnaz Anjum

Ref.number: 34537849
Date: 11 February 2025

Ethical evaluation of research project

Your project, "Resilience but for whom?": A Phenomenological Study on Western-Driven Interventions and Its Detachment from Culturally-Informed Resilience "Sumud" Within Syrian Refugees in Greece" has been ethically evaluated by the Department of Psychology's internal research ethics committee.

After the evaluation The Department of Psychology's internal research ethics committee recommend the project.

Sincerely yours, on behalf of the Committee,

Professor Silje Endresen Reme, Head of Committee
Members of the Department of Psychology's Research Ethics Committee
https://www.uio.no/for-ansatte/enhetssider/sv/psi/psi-eng/internal-ethics-committee/index.html

Postal address:
E-mail:
www.uio.no

Appendix D: SIKT Data Management Plan

DATA MANAGEMENT PLAN

"Resilience but for whom?": A Phenomenological Study on Western-Driven Interventions and Its Detachment from Culturally-Informed Resilience "Sumud" Within Syrian Refugees in Greece

This research project is part of a master's thesis in the psychology master's program of Global-MINDS at Psykologisk institutt (PSI) at the University of Oslo. This research will be conducted by the student Karen Youssef, supervised by Professor Halina Grzymała-Moszczyńska and Associate Professor Gulnaz Anjum. This qualitative research project aims to explore the culturally-informed resilience, "Sumud", within Syrian refugee women and children in Mavrovouni camp in Lesvos, Greece. Through semi-structured interviews with mothers and psychosocial activities with children, this study aims to understand their own definition of resilience, its connection to their past, present, and future, how they pass it on to younger generations, and its alignment with NGO interventions in fostering resiliency.

Disciplines

Social Science

Research responsible institution

Universitetet i Oslo / Det samfunnsvitenskapelige fakultet / Psykologisk institutt

Project duration

20.12.2024 — 31.07.2025

Project Reference Number: 34537849

Scientific purpose

This cultural and social psychological research project aims to understand how different communities define, perceive, and practice resiliency within their own collective and cultural context. Within the context of Syrian refugees, the word "Sumud" is the closest word in the Arabic language to resemble the meaning of resiliency. This research seeks to explore Sumud within Syrian refugees on Lesvos island; which serves as a transitioning point for refugees from their home country into asylum seeking process within the EU. The project will explore this phenomena through semi-structured interviews with Syrian mothers and psycho-social activities with children to address these three main research questions: Q1. How do Syrian

refugee women define, experience, and practice "Sumud" along their journey (before leaving the homeland\ during\ future)? Q2: How do these women pass on Sumud's ideological value to their children and the younger generation? Q3: How do NGOs' resilience-building interventions align with that of the communities' culturally informed resilience?

Utility

Through this recognition, and by portraying the community's voices and own narratives of their strength, we aim to better understand their protective factors and help improve NGOs' interventions tailoring culturally specific tools and resources

Contributors

Gulnaz Anjum gulnaz.anjum@psykologi.uio.no
Supervisor
Halina Grzymała-Moszczyńska halina.grzymala-moszczynska@ignatianum.edu.pl
Supervisor

Does the project involve research on humans?

Yes

Describe the considerations or measures taken to safeguard the integrity and privacy of the participants

This project will remain strictly social psychology-focused and include no diagnostic or clinical aspects. The study will not involve risks; some questions may trigger affective reactions, which the participant has complete freedom not to answer. However, for questions that may trigger affective reactions, the primary researcher/investigator (Karen Youssef) and supervisors (Dr. Gulnaz Anjum & Dr. Halina Grzymała-Moszczyńska) are equipped and trained for such reactions. Dr. Halina Grzymała-Moszczyńska is a psychologist specialized in clinical psychology, cultural psychology, cross-cultural psychology, and psychology of migration with extensive research with refugee mothers and children. Dr. Gulnaz Anjum is also trained in Mental Health and Human Rights Info's (MHHRI) Gender-Based Violence Manual for Women (https://www.hhri.org/) with certification. They are available to provide support for any psychological distress experienced by participants. The researcher Karen received training in Active Listening and Collaborative Intervention in Suicidal Crises as a Suicide Lifeline Operator (https://embracelebanon.org/). In regards to the refugee community, the researcher has previous experience working with refugee women and children through the Lebanese Red Cross. She has also

worked as Special Needs Paraprofessional for an 8-year-old which equips her to handle distressing situations that she might encounter through her activities with the children. The researcher has also interned for three months on Lesvos island at "Parea" community center where she gained

exposure and training in the island's current refugee situation, as well as developed confident interaction and connection with the camp's community. She is also currently working alongside Max Le Roux (Clinical Psychologist) at "Terra Psy" as an interpreter, who will guide on how to ensure participant protection. There are, however, no known harms to the research, but if it comes to emotional outbursts, or if the participant feels unease or discomfort, the researcher is trained to work in these situations and has the required certifications. Moreover, participants will also be given leaflets post-interview, which provides a list of NGOs around the island and within the camp which provides free psychological and emotional support if needed. As with any studies working with interviews, some people may perceive some of the questions as confusing because of their open-ended nature. We will make sure to remove these confusions by reiterating the questions and objectives. Moreover, we will run a mock interview for any additional preparedness. Questions in the interview deal with participants' definition of resilience, the different way in which them and their children experience it throughout their journey, its connection to homeland, culture, and practices, and their experience with NGO's interventions throughout this journey. For children's psychosocial activities, the children will sketch drawings and crafts that expresses their emotions towards different aspects of their life (draw something/someone that makes you happy/angry) to identify their protective and risk factors. The activities will be done under "Terra Psy" organization as part of their series of workshops and alongside a psychologist from the organization with written approval of their caregivers. The participants can withdraw at any time during the study without providing a reason. Their informed consent will be taken, as well as their anonymity will be secured. If they have further questions regarding any aspect of the study, the contact details of the primary researcher and supervisor will be given.

Does the data collection involve children or vulnerable groups?

Yes

Does the project involve animals?

No

Could the project impact the environment, health, or safety?

No

Are third-party data used in the project?

No

Are cultural and indigenous peoples' data used in the project?

No

Is artificial intelligence (AI) used in the project?

No

Does the project involve international cooperation?

Yes

Describe the risk assessments

All necessary precautions have been taken to comply with ethical, legal, and cultural standards for conducting research on Lesvos island and the approval of the NGO in which the psychosocial activities will be taking place is also ensured. Since the confidentiality and anonymity of participants will be strictly maintained throughout the study and written informed consent will be obtained, the research will not cause an specific risk or security challenges towards the participants, or violate any Greek laws and regulations.

Does the data have potential for dual-use applications?

No

Intellectual property rights

The rights to the results of this Master belong to the student and researcher (me), in collaboration with the University of Oslo. This is in line with the University and university college law § 1-5 on academic freedom and responsibility.

What research ethics guidelines apply to the project?

General guidelines for research ethics

Guidelines for Research Ethics in the Social Sciences and the Humanities

Interview Data

Description

The interview data will contain recordings of the women and notes taken (by the interviewer)

Data type

Sound, Text

Language

English, Arabic

Does the data collection include data about people?

Yes

Categories of personal data

Special / criminal offences

Does the data collection include data on vulnerable groups?

Yes

Number of individuals in the dataset

10

Does the data collection include confidential or graded information?

Yes

Are there any other reasons why the data collection needs extra protection?

No

Security class

Confidential

Comment

The data collection and data material is classified as red, the data will be carefully guarded with strict access regulations and will be at all times transferred and stored at a secure server (TSD) as per the University of Oslo's regulations.

Data collection period

01.01.2025 — 15.02.2025

Collection devices

- Sound recorder/dictaphone, institution
- UiO Nettskjema

Data quality

The semi-structured interviews will follow an interview guide, with prompts and guidance questions. This is developed and attached with this application.

Method

Interview, Recording

Description

These will be one-to-one interviews, taken place in spaces where the women feel most comfortable and safe. Participants have the option to deny a recorded interview and still participate in the study by allowing the researcher to take handwritten notes. Otherwise, they will consent for recording the interviews.

Size

100000 MB

Format

docx, m4a

Software

Nettskjema, Dictaphone app, TSD (all relating to UiO) I will analyse the material manually, without the use of a software

Comment

The data will be collected through recordings of individuals using Nettskjema and the UiOs Dictaphone app and process and store data on TSD on its cloud service.

Naming conventions

Folders in TSD: There will be 1 folder, where the 4 sub-folders will have:
1) Audio 2) contact information, personal information, anonymisation key
3) transcriptions and 4) completed anonymised transcriptions

Storage

TSD (Services for Sensitive Data)

Transfer

Unit FileSender

Comment

The data will be collected through recordings of individuals using Nettskjema and

the UiOs Dictaphone app and process and store data on TSD on its cloud service.

Archiving

No

Workshop Data

Description

The workshop data will contain pictures of the children's drawings/sketches/collages,

notes of their statements (taken by the researcher), and audio recordings of the workshops

Data type

Text, Image, Sound

Language

English, Arabic

Does the data collection include data about people?

Yes

Categories of personal data

Special / criminal offences

Does the data collection include data on vulnerable groups?

Yes

Number of individuals in the dataset

10

Does the data collection include confidential or graded information?

Yes

Are there any other reasons why the data collection needs extra protection?

No

Security class

Confidential

Comment

The data collection and data material is classified as red, the data will be carefully guarded with strict access regulations as per the University of Oslo's regulations. The paper-based drawings and sketches will not

involve any name or written information that will identify the participant.

In addition, the data will be pseudonymized and Physically stored in a locked, secure box with the researcher until the end of the project. The workshops will also be audio recorded using via Diktofon and will be transferred and stored at a secure server (TSD) as per the University of Oslo's regulations.

Data collection period
01.01.2025 — 15.02.2025

Collection devices
UiO Nettskjema
Sound recorder/dictaphone, institution

Data quality
The psychosocial workshop will follow a series of sketches, with prompts and guidance questions. The activities will be done under Terra Psy organization as part of their series of workshops and alongside a psychologist from the organization with approval of their caregivers.

Method
Observation, Other, Focus Group, Recording

Description
The workshop will involve paper-based drawings and sketches. It will be led by the organization's psychologist and the researcher. The activity will involve prompts for drawings, observation of activity, and follow-up questions.

Size
100000000 MB

Format
docx, m4a

Software
Nettskjema, Dictaphone app, TSD (all relating to UiO) I will analyse the material manually,

without the use of a software

Comment

The data will be collected through recordings of individuals using Nettskjema and the UiOs Dictaphone app and process and store data on TSD on its cloud service. The drawings will be physically stored in a secure locked box with the researcher.

Naming conventions

Folders in TSD: There will be 1 folder, where the 4 sub-folders will have: 1) Audio 2) contact information, personal information, anonymisation key 3) transcriptions and 4) completed anonymised transcriptions

Storage

TSD (Services for Sensitive Data)

Transfer

Unit FileSender

Archiving

No
